# Jack-of-all-trades, master of none: Postgraduate perspectives on interdisciplinary health research in Australia

**DOI:** 10.1186/1472-6963-7-48

**Published:** 2007-04-04

**Authors:** Gemma E Carey, James A Smith

**Affiliations:** 1 Discipline of Public Health, School of Population Health & Clinical Practice, Faculty of Health Sciences, University of Adelaide, Adelaide, South Australia, Australia; 2 Discipline of Anthropology, School of Social Sciences, University of Adelaide, Adelaide, South Australia, Australia; 3 Discipline of Medicine, School of Medicine, Faculty of Health Sciences, University of Adelaide, Adelaide, South Australia, Australia

## Abstract

**Background:**

Interdisciplinary health research is increasingly perceived as an expectation of research institutions and funding bodies within Australia. However, little consideration has been given to the extent to which this re-orientation has produced a new type of researcher – an interdisciplinary health researcher.

**Discussion:**

As cross-enrolled postgraduate research students, we assert that we do not have an intellectual home. Rather, we must forge a virtual intellectual home through the process of bridging disciplines. In this paper we explain that this virtual home affords us the role of 'interlockers' in future health research. The interlocker role privileges a breadth of understandings across disciplines, rather than a depth in one.

**Summary:**

We conclude by reiterating that there is an undeniable need for interdisciplinary health research, and that the roles and actions of interdisciplinary health researchers need to be better understood and catered for. We therefore call for increased consideration and discussion concerning the future roles and capacities of interdisciplinary health researchers such as ourselves.

## Background

'Interdisciplinary research can be one of the most productive and inspiring of human pursuits – one that provides a format for conversations and connections that lead to new knowledge.'

[1] (Committee on Facilitating Interdisciplinary Research 2004, p1)

For decades academics have endeavored to address the intricacies and complexities of conducting interdisciplinary research [2,3]. Interdisciplinary health research has grown substantially in Australia over recent decades, to the point where it is now an expectation for researchers to work in a collaborative manner, particularly in health research [4]. This increased proclivity for inter-disciplinary health research has extended out of a national and international push for research to encompass a breadth of views and understanding on a given subject [1,4,5]. This push has been fostered by research funding bodies and has led to the formation of 'research clusters', and interdisciplinary journals [6-9]. Arguably, it has resulted in a significant re-orientation of research structure and inquiry. This re-orientation and increased interdisciplinary activity has inevitably given rise to a relatively new phenomenon: the interdisciplinary PhD student. It is worth noting it this juncture that many universities, ours included, do not explicitly seek to recruit or encourage interdisciplinary postgraduate study. However, many academics are encouraged to pursue interdisciplinary research when applying for grant funding. This is part of an increased investment into interdisciplinary activity at broader institutional levels, which has resulted in an implicit encouragement of postgraduate interdisciplinary research.

From the outset we want to provide clarification about what the focus of this paper *is *and *is not*. With increased interdisciplinary activity at all levels, the epistemological differences and difficulties of conducting such research have come under academic scrutiny. Although we acknowledge that this is a concern for interdisciplinary students such as ourselves, this is not a paper on epistemological disparities. The debates and concerns that have been raised in this area have tended to focus somewhat narrowly on the differences between specific disciplines [10-12]. There has been little analysis of the special institutional and intellectual demands of interdisciplinarity in health research, and the special skills and resources required to meet them [13]. In particular, postgraduate student perspectives have almost entirely been absent from that discussion which has occurred. In this paper we wish to appeal to researchers attempting to shape the future of interdisciplinary research, rather than focusing on those who appreciate specific disciplinary frameworks and epistemological conundrums. The purpose of this paper is to create debate and provide inspiration for interdisciplinary postgraduate students to critically reflect on the administrative and intellectual challenges they face. We therefore envisage that this is a starting point of a much broader conversation about how to provide adequate support for students who are engaged in interdisciplinary research.

Relatively little attention has been devoted to cross-enrolled, interdisciplinary PhD students. Whilst the production of such students can be considered an acknowledgement that interdisciplinary work is both growing and imperative, the experience can differ significantly. Due to the avant-garde nature of the interdisciplinary PhD, postgraduate students may be haphazardly accommodated into an existing system that is structured along strict disciplinary lines. While our existence may be considered evidence of the propensity and need for interdisciplinary health research, we suggest that the experience of being a cross-enrolled student often results in one feeling more like a *byproduct *of such research.

The current emphasis on collaboration has led many academics to debate the nuances of pursuing, conducting, and publishing interdisciplinary research [14]. It has been well recognized that such research poses significant challenges in the aforementioned areas of conducting and publishing research [7-9], [15,16]. In this paper we will draw on our experiences as interdisciplinary cross-enrolled postgraduate students in Australia, to facilitate discussion around the role of interdisciplinary health researchers. We call for increased discussion not only around the accommodation of students, but also consideration of our future career trajectories. We will argue that there is both an intellectual and practical niche for researchers such as ourselves [14,17]. However, if we are to fulfill this niche, greater attention must be given to the complexities of being an interdisciplinary health researcher [14]. Increased consideration must also be given to how this new type of researcher will be accommodated in a system, which remains strongly discipline orientated.

This is of particular relevance in Australia due to the current re-structuring of university funding through the introduction of the national Research Quality Framework (RQF). The incorporation of interdisciplinary research into the RQF is crucial, as the ability of the RQF "to deal effectively with cross-disciplinary research will be a crucial determinant of its credibility and ultimate utility" [18, p8]. As of yet there are no clear guidelines concerning the incorporation of interdisciplinary researchers into this framework. The latest draft of the RQF clearly states that the consideration of interdisciplinary research, and early career researchers is critical [19]. This is also of international relevance as the RQF is founded in the respective UK& NZ equivalents, the Research Assessment Exercise and the Performance Based Research Fund [20]. Discussion concerning the future of interdisciplinary health research is therefore vital at this juncture.

## Discussion

### What is interdisciplinary health research?

In this paper we consider interdisciplinary health research to encompass research, which draws on the perspectives of one or more health related disciplines. This includes disciplines within the same paradigm, for example two disciplines that are both based in the social sciences, and disciplines that span across paradigms, such as the social sciences and medical sciences. We will also briefly examine how terms such *as interdisciplinary*, *cross-disciplinary *and *multi-disciplinary*, have been defined, and allude to the subtle differences between them.

Cross-disciplinary research is generally framed and conducted as either 'multi-disciplinary' or 'interdisciplinary' [21,22]. Multi-disciplinary research is where individuals from different disciplines bring a variety of methods and theories to bear on one problem [21,22]. Inter-disciplinary research is where diverse theories and approaches are developed into a singular framework for inquiry [16,21,22]. The interdisciplinary PhD student, however, bears a resemblance to both of these styles, but is reducible to neither. While interdisciplinary health research is a familiar concept to most, an interdisciplinary researcher is not. Previous conceptions of interdisciplinary health researchers may have included academics that draw on a range of research experience, within specific disciplines. That is, researchers who change disciplines at various points in their career. In this paper we draw attention to cross-enrolled PhD students, who are interdisciplinary health researchers from the outset of their career.

### Finding a virtual intellectual home

As cross-enrolled PhD students we are, in fact, in the unique position of embodying the perspectives of multiple disciplines. From our past experiences we have found that we are unable to approach or frame a health problem from the perspective of one discipline. Rather, our approach will always be a hybrid of the multiple disciplines we draw on. Indeed, for us, our roles have emerged out of a perceived health need, rather than a desire to 'create' an interdisciplinary health researcher. We are indeed jacks-of-all-trades, but masters of none. But is this problematic? Well, this perspective tends to advocate breadth over depth. The majority of universities, and research institutions remain organized along strict disciplinary boundaries, despite the increasing forays into interdisciplinary research. Such structuring tends to privilege a nuanced and in-depth understanding of *one *discipline, rather than appreciating a breadth of knowledge across many [1,21]. This leaves the interdisciplinary PhD student in the unsettling position of feeling both encouraged to undertake interdisciplinary health research, but simultaneously lacking an intellectual home.

Whilst interdisciplinary PhD students are faced with a myriad of administrative and intellectual conundrums, all of which we argue require further discussion, in this paper it is the absence of an 'intellectual home' we wish to interrogate. Whilst we may not have a disciplinary home, this in no way connotes that we do not have a disciplinary role. This role is in bridging disciplines, it begins as PhD students, and we suggest is likely to extend throughout our careers.

For researchers of all levels, one of the major obstacles to conducting interdisciplinary health research is finding appropriate researchers from diverse backgrounds to collaborate with. The organization of universities along strict disciplinary lines, in conjunction with disparate physical locations adds to this challenge. The interdisciplinary PhD student begins bridging disciplines the moment they enroll. Having cross-enrolled PhD students breaks down these disciplinary boundaries, affording supervisors the chance to meet other academics and understand the perspective of other disciplines. What may begin as collaboration over one student's project may progress to collaboration in teaching and research. This process of bridging disciplines can represent a 'virtual intellectual home'. This bridging of disciplines is likely to be our intellectual home throughout our careers, not just as PhD students.

Interdisciplinary health researchers have been described as 'interlocutors' [23]. This term suggests that interdisciplinary researchers take part in a shared conversation. Whilst this description is valid, we suggest that our role is more appropriately encapsulated by the term 'interlocker'. Rather than simply taking part in a shared conversation, interdisciplinary health researchers pull multiple perspectives together, and negotiate the tensions and divides of working between disciplines.

### The birth of an *interlocker*

The role of the 'interlocker' privileges a researcher who has a breadth of knowledge in theory, approach, and discourse, rather than intricate knowledge of one discipline. Interdisciplinary health researchers and PhD students are positioned to introduce the discourse and approaches of alternate disciplines. This provides new perspectives for researchers affiliated with singular disciplines, and facilitates the breakdown of disciplinary boundaries. Ultimately, the interdisciplinary researcher is at the forefront of expanding the research imagination [16,24]. In turn, this has the potential to translate health research into policy and practice. In our experiences as PhD students we have already experienced, and demonstrated, our 'interlocker' roles. Our research has necessitated that we think laterally, to overcome the challenges of 'not belonging'. We must recognize the similarities between disciplines, and appreciate and incorporate the unique contributions from each. The most challenging aspect of this work is attempting a synthesis of disciplinary ideas, which is both relevant and accessible to our supervisors' disciplines, despite being the product of two or more.

Our interlocker role also encompasses elements of being a translator. This is of particular relevance to health research. It is frequently acknowledged that health and ill health is best approached in a holistic manner. The causes of ill health lie in social, cultural, environmental and biological factors [25-27]. However, the disciplines that have expertise in these particular areas frequently speak in different dialects [7,17]. These dialects can 'at times sound very much like common language, leading the uninitiated reader to the mistaken conclusion that he or she understands what is being said' [7, p299]. Both at a postgraduate level, and at later career stages, interdisciplinary researchers must contribute their unique understandings by translating these dialects to other disciplines. This is a fundamental concept for legitimating the research role of interdisciplinary researchers.

Naturally, the interdisciplinary health researchers' relationships with disciplines with which they are involved are not always harmonious. We have found in our own work that tensions and conflict often occur, particularly when academics with a strong grounding in a specific discipline attempt to maintain, and police disciplinary boundaries. Despite the push for collaborative research, departments organized around disciplines rather than common goals, can be hostile to researchers who attempt to breakdown the divide. In breaking down disciplinary boundaries an interdisciplinary health researcher can be perceived as threatening, or attempting to undermine foundations of specific disciplines, which operate relatively independently [16]. These tensions, however, can also be beneficial; they can lead to substantial growth in both research and researchers. As PhD students we have learned such skills as tolerance, translation and prudence.

## Summary

The role of interlocker is potentially invaluable, and in light of this we argue that increased consideration and commentary on the role of interdisciplinary PhD students and researchers must occur. The re-orientation of the research sector has led to an ever-increasing prevalence of interdisciplinary PhD students, especially in the area of health. More thought must be given to how research opportunities for these students may be better facilitated, and what types of roles they will have throughout their career. Furthermore, an effort must be made to legitimate these roles in the eyes of those researchers who align themselves to a specific discipline. We suggest that this can be achieved through increased academic discussion and debate around this topic. This is particularly relevant in Australia, during the development of the national Research Quality Framework. During this re-structuring of Australian academic funding, lively international debate concerning the future of interdisciplinary researchers is of paramount importance.

## Competing interests

The author(s) declare that they have no competing interests.

## Authors' contributions

JS conducted a literature search and determined the focus of the paper. GC completed a first draft of the manuscript based on preliminary discussions between both authors. From this point both authors worked equally in refining the concepts, ideas and content of the manuscript. Both authors read and approved the final manuscript.

## Pre-publication history

The pre-publication history for this paper can be accessed here:


